# Maxillofacial Fractures in Southern Hungary: A 15-Year Retrospective Cross-Sectional Study of 1948 Patients

**DOI:** 10.3390/jcm15010280

**Published:** 2025-12-30

**Authors:** Zsolt Rajnics, Olivér Horváth, Viktória Horváth, Parnia Salimian, Gyula Marada, József Szalma

**Affiliations:** 1 Department of Prosthodontics, Dental School, Faculty of Medicine, University of Pécs, Tüzér Str. 1., 7623 Pécs, Baranya, Hungary; 2 Department of Oral Diagnostics, Dental School, Faculty of Medicine, University of Pécs, Tüzér Str. 1., 7623 Pécs, Baranya, Hungary; 3 Department of Oral and Maxillofacial Surgery, Dental School, Faculty of Medicine, University of Pécs, Tüzér Str. 1., 7623 Pécs, Baranya, Hungary

**Keywords:** maxillofacial injuries, mandibular fractures, maxillary fractures, trauma, retrospective study, cross-sectional studies, epidemiology

## Abstract

**Background/objective:** Maxillofacial fractures continue to represent a significant public health issue, with incidence patterns shaped by regional and demographic variables. This study aimed to deliver a comprehensive 15-year epidemiological analysis of maxillofacial trauma cases in southern Hungary. **Methods:** The study included patients who received treatment for maxillofacial trauma at the University of Pécs from 2009 to 2023. Data collected encompassed demographic characteristics, injury etiology, fracture location and complexity, date of injury, presence of alcohol involvement, therapeutic interventions, postoperative complications and reasons, and number of fixation plates removed. Descriptive statistics and odds ratios were calculated, with statistical significance defined as *p* < 0.05. **Results:** Among 1948 patients (69.9% male), a total of 2826 fractures were reported, averaging 1.45 fractures per patient. The most frequently affected age group was 21–30 years; however, a notable increase in cases among the elderly was observed for recent years. Falls accounted for the highest proportion of injuries (44.4%), followed by assaults (28.3%) and traffic accidents (16.8%). Injuries predominantly occurred on weekends, with Saturdays being particularly common. Alcohol consumption was documented in 14.7% of cases. The condyle (27.9%), body (25.7%), and angle (25.0%) were the most common sites of mandibular fracture. The maxillary sinus and zygomatic body were the leading sites of maxillofacial fractures. Conservative treatment was implemented in 54.6% of all cases, whereas surgical intervention was more frequently required for mandibular injuries (76.7%). Plate removal was performed in 15.3% of patients. **Conclusions:** During the study period, the incidence of maxillofacial trauma demonstrated a consistent increase, accompanied by demographic changes indicative of an aging population and a reduction in assault-related cases. Falls—especially among older adults—became the leading cause of injury. These results emphasize the necessity for targeted prevention efforts, geriatric-specific trauma management, and the implementation of health policies tailored to regional needs.

## 1. Introduction

Maxillofacial fractures are among the most frequently encountered traumatic injuries in surgical practice, with significant functional, aesthetic, and socioeconomic implications. Epidemiological studies consistently demonstrate a predominance of young adult males, with road traffic accidents, assaults, and falls as the leading causes; however, their etiology, incidence, and characteristics are highly variable based on time and localization of data collection [1]. Geographic area, culture, ethnicity, social status, and lifestyle differences can all affect these factors, but do not provide a general conclusion that can be used in every circumstance [1,2]. Investigating trends and alterations of maxillofacial traumas are very important: changes in incidence can help governments, healthcare providers, and professionals to improve and plan personal, infrastructural, and financial needs in each service area [3]. Data can be collected on different factors: patients’ sex and age, etiology of trauma, localization of injuries, and treatment options [4].

Over recent decades, the treatment of maxillofacial fractures has undergone a paradigm shift. Open reduction and internal fixation (ORIF) using titanium miniplates and fixation screws has become the standard of care for most mandibular and maxillofacial fractures, offering superior stability, early mobilization, and reduced rates of malocclusion and non-union compared to conservative methods such as mandibulomaxillary fixation (MMF) [5,6,7]. However, ORIF is associated with risks including surgical site infection and trigeminal nerve injury, particularly in complex mandibular fractures [8]. IMF and dental splints remain relevant for select cases, especially in pediatric populations or minimally displaced fractures [9]. Contemporary practice increasingly incorporates minimally invasive and endoscopically assisted techniques, particularly for zygomaticomaxillary complex fractures, which demonstrate comparable functional outcomes and reduced scarring, faster recovery, and improved aesthetic results relative to traditional open approaches [10]. The integration of virtual surgical planning and three-dimensional printing further enhances precision and outcomes in complex cases. Despite these advances, treatment selection must be individualized, balancing fracture characteristics, patient factors, and resource availability [11].

A major limitation of the existing literature is the lack of methodological uniformity among epidemiological studies. Variations in inclusion criteria, age stratification, etiological classification, and outcome measures substantially hinder comparison across studies, some investigating only one-of-a-kind etiology traumas [12,13,14,15] using non-quantified age groups [16,17] or special circumstances [14,15,18]. Non-uniform classification makes data comparison difficult. As factors can change amongst countries and even between regions of a country [2], it is necessary to provide data that is related uniquely to a regional institution in that area. Because of this reasons, the aim of our retrospective cross-sectional study was to analyze the epidemiology of maxillofacial injuries over 15 years in Pécs, southern Hungary an provide research information from the East-Central European region. A retrospective cross-sectional study design was chosen, because we reviewed our former medical records and clinical databases for 2009–2023 and analyzed the distribution of fracture types, patient demographics, and treatments at the time of data collection, without following our treated patients over time [19]. Our further goal was to provide extensively detailed data for better analysis, dividing each investigated criterion as much as possible. Besides the above, the study also investigated daily distribution, role of alcohol, and removal of surgical plates related to maxillofacial fractures.

## 2. Materials and Methods

This retrospective cross-sectional study was conducted at the Department of Oral and Maxillofacial Surgery, University of Pécs Medical School in February 2024. Data collection was approved by the Department of Data Protection, Equal Opportunity and Coordination, University of Pécs Medical School under license KK/263-1/2024.

**Figure 1:**
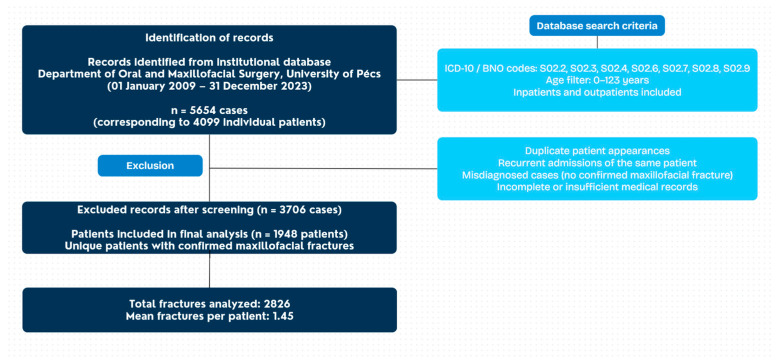
Flowchart illustrating patient identification, screening, exclusion, and final inclusion in the study.

Patients receiving treatment for maxillofacial trauma between 1 January 2009 and 31 December 2023 were included. The collected data included patient sex, age, date of the trauma (day of the week), trauma mechanism (i.e., etiology), influence of alcohol during the trauma, localization and characteristics of the fracture (lower or upper jaw or both, number of fracture lines), the applied therapy, and the reasons for and time of surgical plate removal. Patients were excluded from the analyses if their medical records were incomplete or they had been misdiagnosed with maxillofacial fractures (Figure 1).

The data were retrieved from the electronic clinical records of the patients (both hospitalized and outpatients) who received treatment for maxillofacial fractures in the given period. Our investigation only focused on single-center data collection and did not include patients who passed away during the time of the trauma or before hospital admission to our department. For the institutional database search, a timeframe between 1 January 2009 and 31 December 2023 was set, and the codes for our outpatient and inpatient departments have been provided. The “age” filter was set to between 0 and 123, and WHO (World Health Organization) ICD-10 (*International Classification of Diseases*) codes in Table 1 were used to select cases eligible for the study. After searching the database with the described settings, a portable document format (PDF) file was acquired, displaying each patient with a unique code, including the date of admission and the relevant diagnosis codes. The same patient’s appearances were listed consecutively with the same codes, making it easier to withdraw recurring appearances. The unique codes generated were used to further search each individual patient’s documentation for CT (computed tomography) scans (2009–2015: Philips Brilliance 16 CT (Koninklijke Philips N.V., Amsterdam, The Netherlands)—0.75 mm slice thickness; 2016–2020: Siemens Somatom Definition CT (Siemens AG, Medical Solutions, Erlangen, Germany)—0.75 mm slice thickness; 2021–2023: GE Revolution CT (GE HealthCare Technologies, Inc., Chicago, IL, USA)—0.625 mm slice thickness), radiological findings validated by a licensed radiologist, and final reports after the surgery. All reports were read by the investigating authors, who have evaluated this information and organized the data in an Excel table (Microsoft, Redmond, WA, USA) based on the previously mentioned variables. For each year, a separate worksheet was used, where the investigators could manually add quantified information. Calculation formulas were added to each tab, and the software calculated the total numbers and percentages of each category.

The authors divided patients into 11 age groups: under 10 years old, 11 to 20 years old, 21 to 30 years old, 31 to 40 years old, 41 to 50 years old, 51 to 60 years old, 61 to 70 years old, 71 to 80 years old, 81 to 90 years old, 91 to 100 years, old and over 101 years old. The rationale for age-group categorization in our retrospective cross-sectional study—specifically using the decadal bands 0–10, 11–20, 21–30, up to 91–100, and over 101 years—was primarily to obtain more detailed data, statistical simplicity, interpretability, and broad applicability across a diverse population. These bands ensured adequate sample sizes within each group and allowed for straightforward comparisons of age-related trends, especially when the underlying biological processes were not the primary focus. The use of decadal age bands is advantageous in contexts where the goal is to describe population-level patterns or to compare outcomes across standardized age intervals [20,21].

Occurrence on days of the week was divided into Monday, Tuesday, Wednesday, Thursday, Friday, Saturday, and Sunday. By acquiring daily distribution, the day of the traumatic event was considered.

Regarding etiology, five trauma mechanisms were considered: traffic accidents (with any type of vehicle), trauma by physical assault, fall and falling accidents, and sport and workplace injuries.

Alcohol use was classified as yes (final report stated alcohol consumption), no (final report did not state any alcoholic influence), and not specified (final reports did not mention alcohol consumption or non-consumption) at the time of the injury.

Fractures were classified as symphysis, parasymphysis, body (corpus), angle (angulus), ramus, condylar process (processus condylaris), coronoid process (processus coronoideus), and alveolar process (processus alveolaris) of the mandible. For the maxillofacial bones (excluding the mandible), the following sites were classified: zygomatic body (os zygomaticus), zygomatic arch (arcus zygomaticus), maxillary bone fractures (walls of the maxillary sinus), Le Fort I, Le Fort II, Le Fort III, blow-out fractures, NOE (naso-orbitoethmoid) fractures, nasal bone fractures (os nasale), fronto-orbital fractures, and alveolar process (processus alveolaris) fractures. For the mandible, complexity was classified as fractures with one, two, or more than two fracture lines.

Therapy options were divided into conservative (no indication for surgery, only analgesic or antibiotic or any drug prescription), and surgical options. Surgical options were subdivided to MMF, closed or open reduction.

Surgical follow-up was also investigated: later hospitalizations were checked, acquiring data for plate removals or complications. Only plates placed and removed by the department were considered in this subgroup.

Descriptive statistics were used to summarize patient demographics and clinical characteristics. MedCalc was used to compute univariate odds ratios [22]. A *p*-value < 0.05 was considered statistically significant.

## 3. Results

Over a 15-year span, 4099 patients (a total of 5654 unique cases) were admitted with mandibular and maxillofacial fracture diagnosis to the Department of Oral and Maxillofacial Surgery, University of Pécs, Medical School. After checking the abovementioned criteria, duplicated and misdiagnosed cases were removed. A total of 1948 patients with 2826 maxillofacial fractures (equivalent to 1.45 fractures per patient) received treatment in the given timeframe by the department (Figure 1). Detailed year-by-year data are provided in the Supplementary Materials (Supplementary Table S1).

### 3.1. Distribution of Patients by Year, Sex, and Age

**Figure 2:**
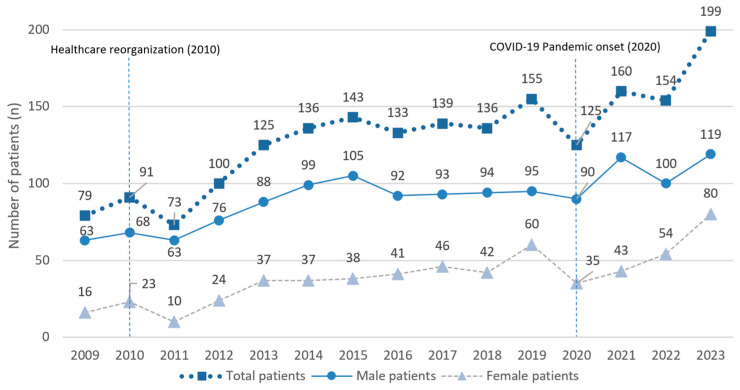
Annual number of patients treated for maxillofacial fractures between 2009 and 2023, stratified by sex. Vertical dashed lines indicate major healthcare reorganization (2010) and the onset of the COVID-19 pandemic (2020).

Figure 2 shows the distribution of patients (total number and sex) for each year between 2009 and 2023. A steady increase in patient numbers can be seen on the line chart, with double the patients treated comparing the early and final years of the studied frame. Overall, 69.92% of patients were male (*n* = 1362) and 30.08% were female (*n* = 586).

**Figure 3:**
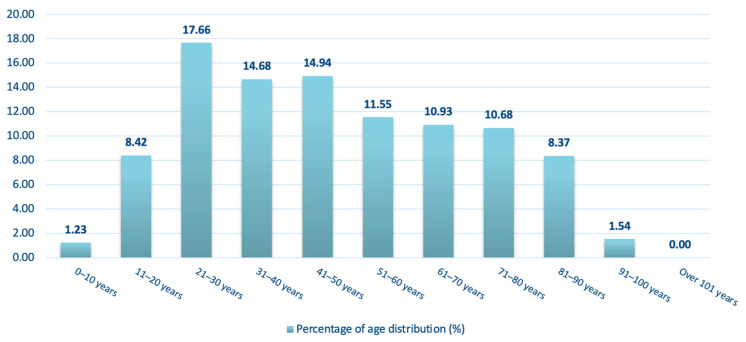
Age distribution of patients treated for maxillofacial fractures between 2009 and 2023. Values represent the percentage of patients within each age group.

**Figure 4:**
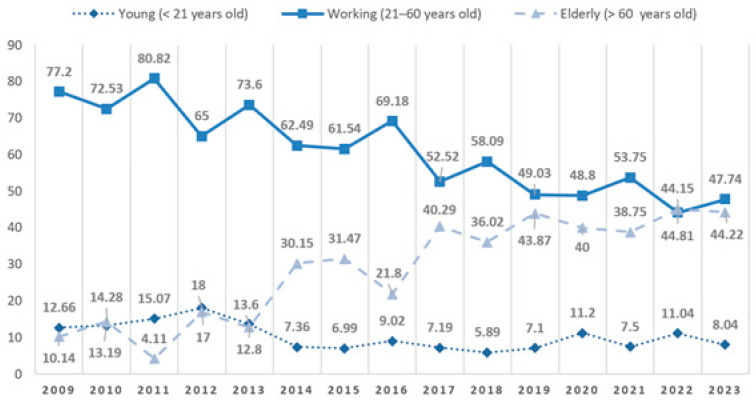
Distribution of young (<21 years old), working (21–60 years old), and elderly (>60 years old) patients treated for maxillofacial fractures between 2009 and 2023. Values represent the percentage of patient groups within a given year.

Age distribution can be seen in Figure 3. Most patients were young and middle-aged adults, with the highest number of cases observed in the 21–30-year age group (*n* = 344, 17.66%). High case numbers were also noted in the 31–40-year (*n* = 286, 14.68%) and 41–50-year (*n* = 291, 14.94%) groups. A slow decline in case numbers was noted with increasing age thereafter, with 225 patients aged 51–60 years (11.55%), 213 aged 61–70 years (10.93%), and 208 aged 71–80 years (10.68%). A sharp decline can be seen between the 81–90- (*n* = 163, 8.37%) and the 91–100-year-old group (*n* = 30, 1.54%). Pediatric (0–10 years; *n* = 24, 1.23%) patients accounted for only a small fraction of the study population. No patient over 101 years old suffered maxillofacial trauma in the study period (oldest patient in our researched period was 100 years, 11 months old). Comparing young (<21 years old), working (21–60 years old), and elderly (>60 years old) patients, we can see changes in their distribution (Figure 4).

### 3.2. Occurrence of Maxillofacial Injuries and Days of the Week

**Figure 5:**
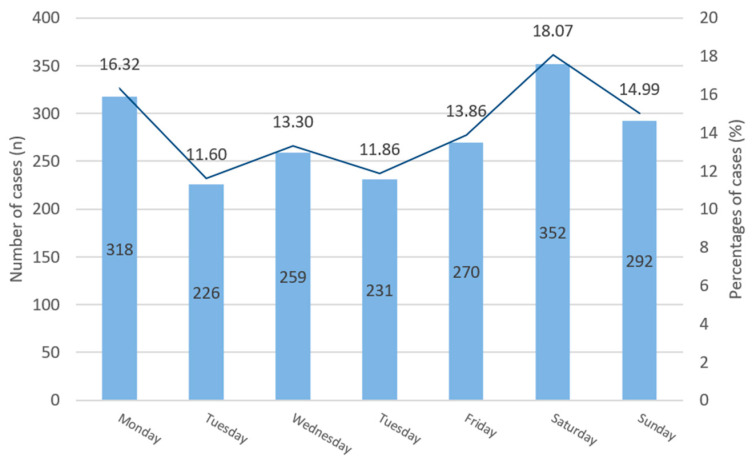
Occurrence on days of the week of maxillofacial injuries in columnar display with number of cases (*n*) (numerically inside each column) and proportional representation as percentages (%) indicated by the overlaid line (numerically above line).

Figure 5 shows the daily distribution of maxillofacial injuries presenting to the department. Many patients received primary treatment by the Department of Emergency Medicine or Department of Traumatology. Admission to the Maxillofacial Division could be delayed by one or more days. To unify the data, we recorded the day of injury rather than the admission to our department. Our records show that most cases happened on Saturday (*n* = 352, 18.07%), followed by Monday (*n* = 318, 16.32%) and Sunday (*n* = 292, 14.99%). These three days represented almost half of all cases seen (49.38%). Tuesday–Thursday (226 (11.6%) and 231 (11.86%) cases) and Wednesday–Friday (259 (13.3%) and 270 (13.86%) cases) had similar numbers of cases. Comparing the busiest and least busy days, there was a 1.6-fold higher chance (OR = 1.68; 95% CI (confidence interval): 1.40–2.01; *p* < 0.001) for a fracture patient to be admitted on Saturday than on Tuesday.

### 3.3. Distribution by Etiology and Alcohol Consumption

**Figure 6:**
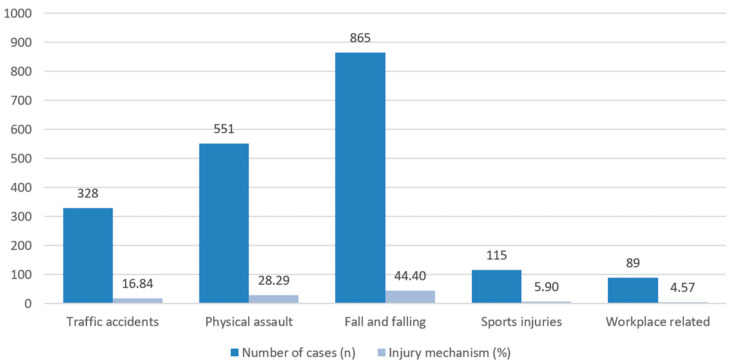
Distribution of maxillofacial injuries based on injury mechanism.

**Figure 7:**
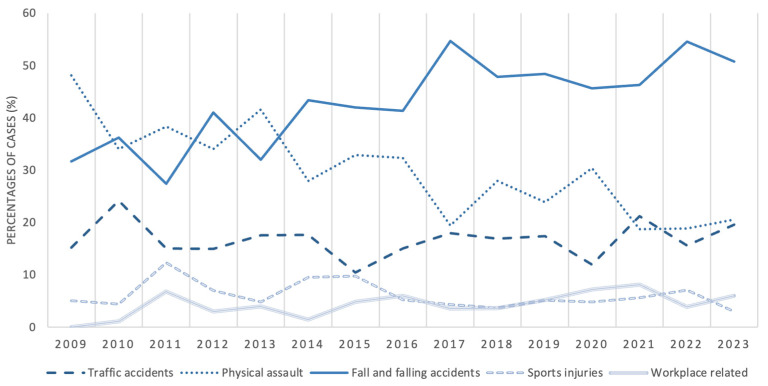
Alteration of etiological factors during the investigated timeframe.

Regarding etiology, we considered five causes of trauma in our research. Traffic accidents with any kind of vehicle (cars, bikes, and similar transportation, apart from vehicles used for work (forklift, trolley, and similar machinery)), trauma and injuries by physical assaults, fall and falling accidents, sports injuries, and workplace-related injuries were considered. Our results are shown in Figure 6 and with detailed data alterations in Figure 7. In most cases, falls and falling accidents were the cause of maxillofacial injuries (*n* = 865, 44.4%), followed by physical assault (*n* = 551, 28.29%) and traffic accidents (*n* = 328, 16.84%). The occurrence of physical assault (OR = 0.49; 95% CI: 0.43–0.56; *p* < 0.001) or traffic accidents (OR = 0.25; 95% CI: 0.22–0.29; *p* < 0.001) was significantly less frequent than fall-related fractures. In about one in ten cases, sports (*n* = 115) and workplace related injuries (*n* = 89) were the reasons for maxillofacial trauma (10.47%).

**Figure 8:**
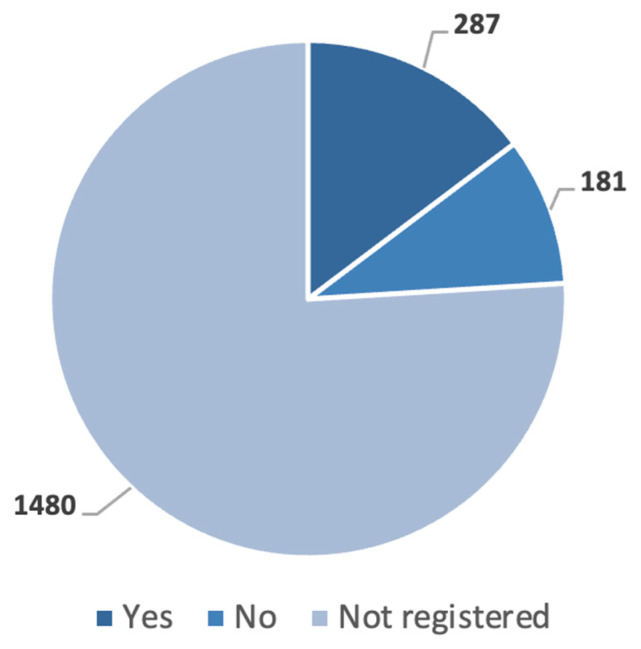
Distribution of maxillofacial injuries according to alcohol consumption (number of cases (*n*)).

Consumption of alcohol related to maxillofacial traumas can be seen in Figure 8. In 75.97% of all cases (*n* = 1480) final reports did not mention alcohol consumption or non-consumption, 14.73% of reports (*n* = 287) noted the patient was under the influence, while 9.29% excluded any alcohol usage (*n* = 181).

### 3.4. Characteristics of Mandibular Fractures

**Figure 9:**
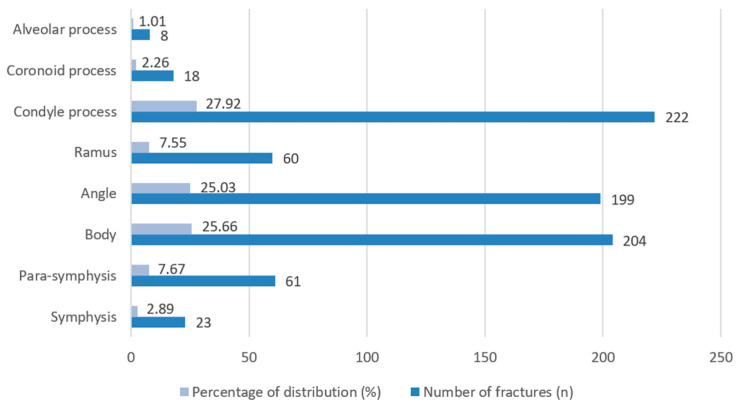
Distribution of mandibular fractures.

Regarding the anatomical distribution of mandibular fractures, the condylar process was the most frequently affected site, accounting for 27.92% of all mandibular fractures (*n* = 222) (Figure 9). This was followed by fractures of the mandibular body (*n* = 204, 25.66%) and angle (*n* = 199, 25.03%), which together with condylar injuries comprised nearly 80% of all mandibular fracture cases. Less commonly involved regions included the parasymphysis (*n* = 61, 7.67%) and ramus (*n* = 60, 7.55%), while fractures of the symphysis (*n* = 23, 2.89%), coronoid (*n* = 18, 2.26%), and alveolar processes (*n* = 8, 1.01%) were rare, representing only a small proportion of cases.

**Figure 10:**
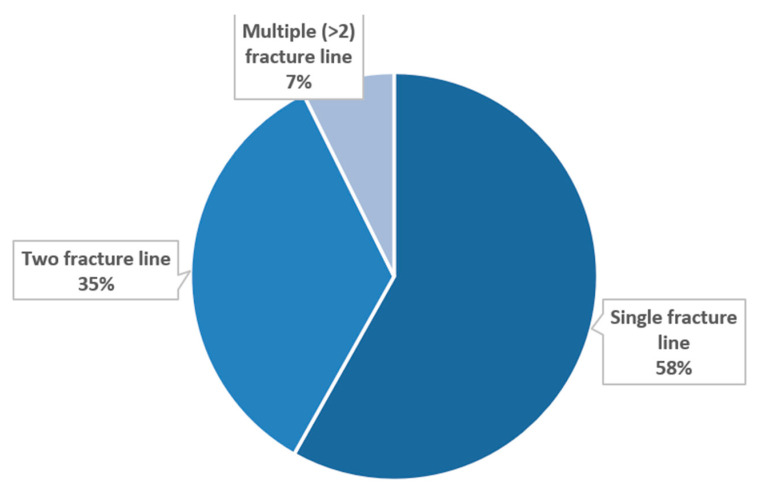
Complexity of mandibular fractures.

Examining the complexity of mandibular traumas, injuries with single fracture lines were the most common (*n* = 324, 58.17%), followed by two fracture lines (*n* = 192, 34.47%) and then multiple (>2) (*n* = 41, 7.36%) fracture line cases. The numerical distribution of complexity can be seen in Figure 10.

### 3.5. Characteristics of Maxillofacial Fractures

**Figure 11:**
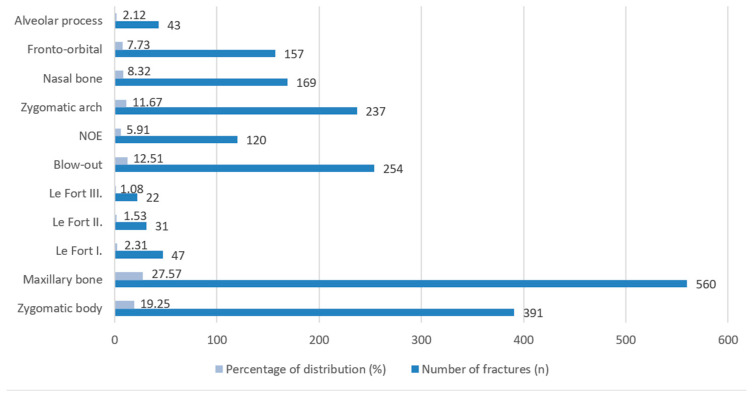
Distribution of maxillofacial fractures.

Regarding fracture localization, the maxillary bone was the most frequently affected site, accounting for 560 fractures (27.57%), followed by fractures of the zygomatic body (*n* = 391, 19.25%) (Figure 11). Orbital blow-out fractures were also common, representing 254 cases (12.51%), while fractures of the zygomatic arch comprised 237 cases (11.67%). Nasal bone fractures (*n* = 169, 8.32%) and fronto-orbital fractures (*n* = 157, 7.73%) were observed less frequently. NOE fractures accounted for 120 cases (5.91%). Le Fort fractures were relatively rare: Le Fort I (*n* = 47, 2.31%), Le Fort II (*n* = 31, 1.53%), and Le Fort III (*n* = 22, 1.08%). Alveolar process fractures were the least common, occurring in 43 cases (2.12%).

### 3.6. Characteristics of Therapy for Mandibular and Maxillofacial Fractures

**Figure 12:**
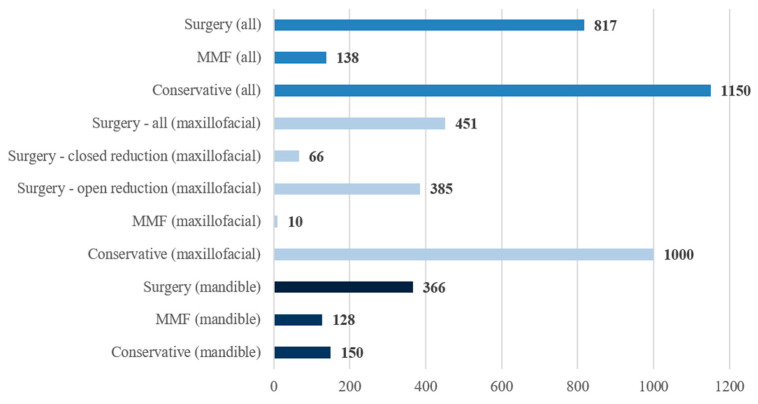
Distribution of therapy options (number of cases (*n*)).

The distribution of therapy options can be seen in Figure 12. In 1150 cases (54.63%), conservative treatment was selected, antibiotic and analgesic treatment were ordered, and patients did not undergo any surgical intervention. Overall, 38.81% of the patients underwent surgical treatment (*n* = 817), while in 6.56% of cases MMF was performed (*n* = 138). Significant differences were however found between mandibular and maxillofacial fractures. While in 76.7% of lower jaw injuries, surgical and MMF treatment was selected, only 31.56% of maxillofacial injuries underwent surgical treatment or by MMF.

### 3.7. Distribution of Surgical Fixation Plate Removal by Number and Cause and Postoperative Complications

After the patients presented with trauma, any later hospitalization episodes were registered. Only fixation plates placed and removed by our department were considered in this subgroup. A total of 680 patients received surgical fixation plates, of which in 104 cases the plates were removed (15.29%), whereas in 576 (84.71%) cases, the plates remained in place.

There were 119 postoperative complications that led to plate removal. These included inflammatory conditions such as cysts, fistulas, bone necrosis (*n* = 29), surgical plate exposure (*n* = 13), combined plate exposure with inflammation (*n* = 10), and sensory loss (*n* = 4). Mechanical complications were rare, with plate or screw fractures occurring in three cases. Additional interventions included sequestrectomy (*n* = 3) and reposition surgery (*n* = 2). Further treatments most commonly involved surgical extraction of third molars (*n* = 13) and other teeth (*n* = 6), followed by bone augmentation or bone regeneration procedures (each *n* = 4) and preprosthetic surgeries (*n* = 2). In several cases, treatment was performed at the patient’s request (*n* = 16) or for unspecified reasons in the final reports (*n* = 10). The sum of cases was higher than the number of plates removed, because in some cases more complications could be observed for a single surgical plate.

Immediate postoperative complications were identified in 104 cases. The most frequently observed complications were inflammatory conditions (*n* = 40). Sensory disturbances were documented in 14 cases, while plate loosening and MMF complications occurred in 12 cases each. Other notable postoperative complications included pain (*n* = 10), diplopia (*n* = 9), and occlusal disturbances (*n* = 7).

## 4. Discussion

In this retrospective study, 1948 patients received treatment at our department in the 15-year timeframe observed. From 2011, a clear increase in annual numbers of patients was seen. This might be the direct result of the healthcare reorganization in Hungary that started in 2010, which rearranged regional healthcare areas, increasing territorial obligation of our department as well. This change occurred in the second year of our 15-year investigated time period, providing at least 13 years of data in the same territorial healthcare system. Patient numbers remained constant in the following years, with a one year decrease in 2020 because of COVID-19 (coronavirus disease 2019). The pandemic hindered and changed social behavior, mobility, and healthcare access, which resulted in the decrease in patient numbers and changes in etiology patterns seen in our results. The sudden increase in the last examined year could be explained by the aftermath of the pandemic, as 2023 was the first year in Hungary without any governmental restriction and regulation.

Our findings show a male/female ratio of 2.3:1, which is similar to the literature, which shows a male dominance related to maxillofacial injuries [1,23,24,25]. Comparing the first five years to the final five years, we can see an increase in the number of female patients: on average, there were 3.74-fold fewer female patients (2009–2013) compared to the recent ratio of 2.04 (2019–2023).

In this work, the most common age for maxillofacial injury was 21 to 30 years (17.66%). Alterations in numbers of young patients decreased and kept steady in recent years, the distribution of elderly patients drastically increased, while active-age patients decreased [17,26,27]. At its peak in 2011, 80.82% of patients were from the working age group, while only 4.11% of patients were considered elderly. In 2023, these numbers showed almost equal proportions, while in 2022 there were slightly more cases in the elderly group compared to the working group (44.81% to 44.15%). Following pattern can be a reason for increased female ratio in recent years in our study. An increase in the elderly population in Hungary (and in developed countries) is well documented. The life expectancy of women is higher compared to men, which results in more elderly female patients [28,29].

Most traumas happened on days related to weekends, as most injuries occurred on Saturdays, Mondays, and Sundays. Weekends had a higher incidence of trauma [30,31], while other reports show no significant change over the days of the week, explained by local trauma care patterns [32].

As seen in Table 2, etiological factors of maxillofacial traumas are highly dependent on geographic localization (including country and regions).

As our data show, injuries related to traffic accidents, sports, and the workplace remained stable. The pattern of falls and falling and physical assault traumas changed from 2014: the differences are well separated, with fall injuries as our main etiological factor to present, which can be correlated with age group changes [38] (Figure 6). The same pattern can be seen between the decrease in active-age patients and physical assault-related injuries. The decrease in injuries related to interpersonal violence is also shown in the HCSO (Hungarian Central Statistical Office) statistics for registered crimes: between 2009 and 2022: crimes related to violence decreased by 46.2% in Hungary [39]. Our research demonstrated a 57.2% decrease in these injuries. In 2020, the increase in physical assaults and decrease in traffic-related accidents could be related to the COVID-19 pandemic [40,41]. During lockdowns, people traveled less (or were not allowed to) and stayed home more, which resulted in increased domestic violence based on HCSO statistics [39,42]. Similar data can be seen in the literature [43,44,45].

In 75.97%, final reports did not include any medical data on alcohol consumption or non-consumption. In 14.73% of cases, we found a correlation between maxillofacial injuries and alcohol influence. Lee and al. reported similar a percentage among fractured patients (18%) [46], while other studies indicated a lower percentage on alcohol consumption (6.6% in traffic accidents) [12]. The role of alcohol is highly variable by region and country, but still plays a critical role in many injury types [47]. Limiting the trueness of this data, blood alcohol measurements are not used routinely in every trauma case. In cases involving traffic accidents and workplace-related accidents, blood tests are required by law and ordered by law enforcement agencies, but in cases of falls and falling or sports-related accidents, these are not uniform diagnostic steps. Patients could also be victims of race, ethnicity, and sex inequities in blood alcohol testing after the event of trauma. The proportion and severity of alcohol-related accidents could be higher, remaining hidden due to differences in diagnostics and procedures [48,49].

Mandibular fractures were most frequently located at the condylar process, the body, and the angle of the mandible. These three sites accounted for nearly 80% of all mandibular fractures, which is consistent with other studies highlighting the condyle and angle as common fracture sites due to their anatomical vulnerability during traumatic impacts [50,51]. Less frequently observed were fractures involving the alveolar process, coronoid process, and symphysis, all contributing to a smaller portion of our results [52,53]. These findings underscore the distribution pattern typically associated with blunt trauma and fall-related injuries, especially in older populations [54]. In terms of fracture complexity, single-line mandibular fractures were most prevalent. The predominance of simple fractures suggests that most traumatic events involved moderate force, though the notable proportion of complex cases may reflect high-energy injuries, such as those from traffic accidents or physical assaults [35,55].

In terms of fractures of the maxillofacial bones (excluding the mandible), the maxillary sinus walls and the zygomatic complex were the most affected sites. Le Fort fractures were present in 4.92% of cases, indicative of severe trauma typically associated with high-impact mechanisms. This distribution aligns with similar retrospective studies in both European and international research [56,57,58].

Our findings on treatment options revealed that over half of the cases were managed conservatively without the need for surgical intervention. This approach is especially common in less or non-displaced fractures or in cases involving elderly patients with multimorbidity [36,59]. A clear contrast was noted between mandibular and non-mandibular fractures: while most mandibular fractures were treated surgically or with MMF, maxillofacial fractures were more often managed conservatively [37,60].

Surgical plate removal was seen in only 104 (15.29%) cases out of the 680 patients. This low percentage suggests either minimal postoperative complications or patient preference against undergoing additional surgery [61]. Nevertheless, regular follow-up and monitoring of retained plates remain crucial to identify late complications, such as infection or surgical plate failure. The observed pattern of complications requiring plate removal aligns with previous reports indicating that early complications are typically related to infection, exposure, or pain, while late complications may arise from chronic discomfort or delayed infection [62]. Notably, infection and plate exposure remain the most frequent indications for hardware removal, consistent with findings from large retrospective cohorts and systematic reviews [62,63]. The anatomical location of the plate is a significant determinant of removal risk. Plates placed in the mandible, particularly in the angle and symphyseal regions, are associated with higher rates of infection and subsequent removal, as demonstrated by multivariate analyses and retrospective reviews [64]. Additionally, patient factors such as smoking and occupation have been shown to increase the likelihood of plate-related complications and removal [62,63].

Several limitations of this study should be acknowledged. The period under review is long enough to encompass the transformation of the healthcare system and territorial care obligations, which in our case happened in Hungary in 2010. These changes may influence the maximum number of cases. COVID-19 happened during the reviewed period, which acted as a temporal confounder, possibly limiting direct longitudinal comparisons and may explain transient deviations in etiology and patient numbers. Our investigation included single-center data collection, possibly not showing every patient who passed away during trauma, before hospital admission, or in other departments. Our findings primarily reflect the epidemiology of survivors who reached definitive maxillofacial care in our department. Due to differences in diagnostics and procedures, not using routinely blood alcohol level measurements in every trauma case could lower the role of alcohol in trauma cases, hiding the real, accurate numbers for statistical purposes. Our study did not address the effects of drugs, narcotics, or other mind-altering substances. Our goal was to represent clinical trauma presentations rather than to highlight underlying psychosocial determinants.

Functional outcomes such as occlusion, temporomandibular joint function, and quality of life were beyond the scope of our retrospective epidemiological study. The study focused on epidemiological and anatomical patterns, and we suggest prospective studies to address functional and patient-reported outcomes.

## 5. Conclusions

Besides the study limitations (single-center data collection and regional (not nationwide) research), this 15-year retrospective analysis offers a comprehensive overview of epidemiology, etiology, anatomical distribution, and treatment modalities of maxillofacial fractures.

Based on our findings, falls, particularly among the elderly population, emerged as the dominant cause of injury, mirroring demographic aging and a rising proportion of female patients. Mandibular fractures, especially at the condyle, body, and angle, were the most prevalent, with single-line fractures being the most typical. While conservative treatment remained a common treatment option for most facial injuries, surgical management was frequently required for mandible trauma cases.

These results emphasize the urgent need for tailored trauma prevention strategies and regionally adapted health policies and call attention to the importance of integrating epidemiological data with social and behavioral insights. Future research should investigate the influence of alcohol consumption and psychosocial stressors, particularly during times of crisis, to better inform public health interventions aimed at mitigating facial trauma risk.

## Figures and Tables

**Table 1:** ICD-10 and BNO (Betegségek Nemzetközi Osztályozása (Hungarian version of ICD)) codes used for patient selection in this study.

ICD-10 Code (WHO)	BNO Code (Hungarian)	Description
S02.2	S0220	Fracture of nasal bones
S02.3	S0230	Fracture of orbital floor
S02.4	S0240	Fracture of malar and maxillary bones
S02.6	S0260	Fracture of mandible
S02.7	S0270	Multiple fractures involving skull and facial bones
S02.8	S0280	Fractures of other skull and facial bones
S02.9	S0290	Fracture of skull and facial bones, part unspecified

**Table 2:** Main etiological factors of maxillofacial traumas.

Author(s) Year of Publication	Number of Patients (*n*)	Country	Main Etiological Factors (%)
Boffano et al., 2015 [2]	3396	Multicenter (Europe)	Physical assaults (39%) Falls and falling accidents (31%) Sport injuries (11%)
Bonavolontà et al., 2017 [16]	1720	Italy	Traffic accidents (57.1%) Physical assaults (21.7%) Fall and falling accidents (14.2%)
Gugliotta et al., 2024 [17]	3424	Italy	Physical assaults (27.4%) Traffic accidents (26.2%) Fall and falling accidents (25.2%)
Spallaccia et al., 2022 [18]	603	Italy	Traffic accidents (36%) Fall and falling accidents (27%) Sport injuries (13%) and physical assaults (13%)
Martinez et al., 2014 [26]	1736	USA	Physical assaults (29.7%) Traffic accidents (29.6%) Fall and falling accidents (22.1%)
Kapoor et al., 2012 [30]	1000	India	Physical assaults (53.8%) Traffic accidents (40.4%) Fall and falling accidents (3.3%)
Conceição et al., 2018 [33]	3262	Brazil	Physical assaults (81.8%) Traffic accidents (11.4%) Fall and falling accidents (4.9%)
Bataineh, 2024 [34]	481	Jordan	Traffic accidents (62.1%) Physical assaults (15.2%) Fall and falling accidents (15%)
Oruç et al., 2016 [35]	283	Turkey	Physical assaults (36.7%) Traffic accidents (32.9%) Fall and falling accidents (29.3%)
Brucoli et al., 2019 [36]	1406	Multicenter (Europe)	Physical assaults (38%) Fall and falling accidents (30%) Traffic accidents (14%)
Kanala et al. 2021 [37]	1112	India	Traffic accidents (70%) Fall and falling accidents (19%) Physical assaults (9%)

## Data Availability

Data are available on request from corresponding author.

## Supplementary Materials

The following supporting information can be downloaded at: https://www.mdpi.com/article/10.3390/jcm15010280/s1, Table S1: Detailed annual dataset included in this retrospective study between 2009 and 2023. The tables provide year-by-year information including demographic data, fracture characteristics, treatment modality, and related clinical parameters used for the statistical analyses presented in the manuscript.

## Abbreviations

The following abbreviations are used in this manuscript.

ORIF	Open reduction and internal fixation
MMF	Mandibulomaxillary fixation
KK	Klinikai Központ (Clinical Center)
WHO	World Health Organization
ICD	International Classification of Diseases
PDF	Portable Document Format
CT	Computed tomography
USA	United States of America
BNO	Betegségek Nemzetközi Osztályozása (Hungarian version of ICD)
NOE	Naso-orbito-ethmoidal
OR	Odds ratio
CI	Confidence interval
COVID-19	Coronavirus disease 2019
HCSO	Hungarian Central Statistical Office
